# Adverse effects of vaping on human health: a brief review of mechanisms and long-term consequences

**DOI:** 10.2478/aiht-2026-77-4084

**Published:** 2026-09-25

**Authors:** Borys Fylenko, Ivan Starchenko, Nataliia Roiko

**Affiliations:** Poltava State Medical University, Department of Pathological Anatomy and Forensic Medicine, Poltava, Ukraine

**Keywords:** adolescent health, cardiovascular system, electronic cigarettes, harm reduction, respiratory system, dišni sustav, elektroničke cigarete, srčanožilni sustav, smanjenje štete, zdravlje mladih

## Abstract

Vaping, often advertised as a safe alternative to traditional tobacco smoking, has recently been subjected to active scientific research due to its potential adverse effects on human health. This narrative review synthesises selected recent literature to provide a comprehensive view of the systemic risks associated with the use of electronic cigarettes. It lists key chemical components (propylene glycol, vegetable glycerine, nicotine, and flavourings) and thermal decomposition products of vape aerosol (formaldehyde, acrolein, propylene oxide, glycidol) and evaluates their multi-systemic toxic effects and clinical manifestations, as well as specific effects on the respiratory, cardiovascular, immune, and nervous systems. Current data suggest that vaping can lead to a wide range of acute and chronic diseases, including the e-cigarette or vaping-associated lung injury (EVALI) and obliterative bronchiolitis and increase the risk of developing oncological, cardiovascular, and neurological diseases. Particularly sensitive is the developing adolescent brain. A growing body of evidence indicates that vaping is not "less harmful" but merely "differently" harmful and calls for further long-term research and strengthened public health regulation.

## Abstract (hr)

Vaping, koji se često promiče kao sigurna alternativa tradicionalnom pušenju duhanskih proizvoda, u novije je vrijeme predmet intenzivnih znanstvenih istraživanja zbog mogućih štetnih učinaka na ljudsko zdravlje. Cilj je ovoga narativnog pregleda odabrane novije literature dati kratki uvid u sistemske rizike povezane s ključnim kemijskim sastavnicama e-cigareta (propilen-glikolom, biljnim glicerolom, nikotinom i aromama) i proizvodima s toplinskom razgradnjom aerosola (formaldehidom, akroleinom, propilen-oksidom i glicidolom), posebice na njihove toksične učinke na dišni, srčanožilni, imunosni i živčani sustav. Dosadašnji podaci upućuju na to da vaping može dovesti do širokog raspona akutnih i kroničnih bolesti, uključujući specifičnu akutnu ozljedu pluća (tzv. EVALI) i obliterirajući bronhiolitis (*bronhiolitis obliterans*), te povećati rizik od razvoja onkoloških, kardiovaskularnih i neuroloških bolesti. Posebno je osjetljiv mozak adolescenata u razvoju. Sve je više dokaza da vaping nije „manje štetan“, nego samo „štetan na drugačiji način“, što upućuje na potrebu za daljnjim dugoročnim istraživanjima i snažnijom regulacijom u području javnog zdravstva.

Electronic cigarettes, also known as vapes, were developed and introduced to the market as a tool for “harm reduction” from tobacco smoking. Their main difference from traditional cigarettes is the absence of tobacco combustion, which, it was claimed, eliminates the formation of carcinogenic tars and other combustion products (1). This narrative quickly took hold in the public consciousness, and vaping positioned itself as a safer alternative or an effective means of quitting smoking (2, 3). Attractive flavours, a pleasant vapor aroma, and a deceptive sense of safety have soon led to its widespread adoption and popularity, especially among adolescents and youth, triggering a new public health crisis (4, 5).

Yet, despite marketing assurances, vapes have come under intense scientific and medical scrutiny, raising deep concern about potential adverse health effects as initial data began to reveal significant risks (6,7,8). A growing number of clinical cases and epidemiological studies suggested that vaping was not harmless but could cause specific damage to organs and systems not observed with traditional smoking (9, 10).

The aim of this narrative review is to synthesise and critically analyse the available scientific literature on the adverse effects of vaping on human health by focusing on the unique mechanisms of toxicity and clinical consequences specific to electronic cigarette use.

## METHODOLOGY

For this review we searched the major scientific databases, including PubMed, Scopus, and Google Scholar, using structured combinations of operators (AND, OR) and keywords directly aligned with the core thematic sections (Table 1).

**Table 1:** Search key words by thematic units

**Chemical composition and thermal degradation**	(“electronic cigarettes” OR “vape aerosol” OR “e-liquids”) AND (“propylene glycol” OR “vegetable glycerin” OR “thermal degradation” OR “formaldehyde” OR “acrolein” OR “propylene oxide” OR “glycidol” OR “heavy metals”);
**Pulmonary pathomorphology and immune response**	(“vaping” OR “e-cigarette”) AND (“EVALI” OR “vitamin E acetate” OR “obliterative bronchiolitis” OR “popcorn lung” OR “lipoid pneumonia” OR “neutrophil dysfunction” OR “actin microfilaments”);
**Cardiovascular and endothelial dynamics**	(“electronic nicotine delivery systems” OR “vaping”) AND (“endothelial dysfunction” OR “nitric oxide” OR “ICAM-1” OR “S100A8” OR “arterial stiffness” OR “thrombogenicity”);
**Neurodevelopment and cognitive impact**	(“vaping” OR “nicotine salts”) AND (“adolescent brain” OR “prefrontal cortex” OR “hippocampus” OR “cognitive impairment” OR “executive function”);
**Harm reduction and comparative risk**	(“e-cigarettes”) AND (“harm reduction” OR “smoking cessation” OR “dual use” OR “lung cancer risk” OR “former smokers”).

To ensure quality and to avoid arbitrary aggregation, source selection followed structured qualitative screening. The initial search yielded 280 records published between 2019 and 2025 of which 160 were excluded as non-peer-reviewed reports, commercial/industry-funded studies with clear conflicts of interest, duplicate datasets, and studies focusing purely on marketing/economic aspects rather than biological and health effects. The remaining 120 full-text publications were evaluated for methodological rigor and topical relevance to finally obtain 52 articles (primary experimental studies, clinical trials, and meta-analyses).

## CHEMICAL COMPOSITION OF VAPE AEROSOL AND MECHANISMS OF TOXICITY

### Main components of e-liquids and harmful products of their heating

Vape liquids (e-liquids) typically consist of four main components: vegetable glycerine (C_2_H_8_O_3_), propylene glycol (C_2_H_8_O_3_), flavourings, and, optionally, nicotine (11, 12). Vegetable glycerine is a viscous, oily, and sweet liquid used in the food industry as the E442 additive. In e-liquid, it is responsible for producing large amounts of vapor. Propylene glycol is a colourless liquid with a slightly sweet taste and a syrupy consistency. It acts as a carrier for flavourings and nicotine and is responsible for the “throat hit”.

A common misconception is that these components are completely harmless because they are used in the food and pharmaceutical industries (13). It fails to account for the critical difference in their delivery into the body. In food products, propylene glycol and vegetable glycerine are consumed orally and metabolised through the digestive system. In vaping, they are subjected to thermal stress at high temperatures, reaching 100–300 °C, depending on the device design. This process, known as thermal degradation, leads to the formation of new toxic byproducts that are not present in the initial unheated liquid (Table 2) (14, 15).

**Table 2:** Main toxic substances formed during e-liquid heating and their adverse effects

**Chemical compound**	**Parent component / Origin**	**Chemical formula**	**Primary pathophysiological impact & target systems**	**Toxicity / Carcinogenicity classification**	**Ref.**
Formaldehyde	Propylene glycol and glycerine thermal degradation	CH_2_O	Mucosal irritation, central nervous system impairment (cognitive/memory deficits), cellular and DNA damage	Known human carcinogen (Group 1 IARC)	(11, 14, 16, 44)
Acrolein	Vegetable glycerine thermal decomposition	C_3_H_4_O	Severe bronchial and ocular irritation, toxic damage to cardiac tissue and airway epithelium	Potent respiratory and cardiac toxicant	(11, 16, 18)
Propylene oxide	Propylene glycol heating	C_3_H_6_O	DNA adduct formation, epithelial damage, systemic toxicity	Likely human carcinogen	(17, 29)
Glycidol	Glycerine thermal degradation	C_3_H_5_O_2_	Mutagenic activity, oxidative DNA damage	Carcinogenic compound	(17, 18)
Heavy metals (Ni, Cd, Cr, Pb)	Degradation of heating coil/elements	Ni, Cd, Cr, Pb	Systemic inflammation, endothelial dysfunction, increased long-term oncological risk	Toxic metals / carcinogens	(20)
Nicotine (when present)	Concentrated e-liquid additive / salts	C_10_H_14_N_2_	Adrenaline release, acute hypertension, tachycardia, endothelial dysfunction, hippocampal alteration	Potent psychoactive / cardiovascular agent	(12, 30, 33, 35, 40)

One of the most important is formaldehyde (CH_2_O), a known carcinogen used as a disinfectant. Another harmful compound, formed by the thermal decomposition of glycerine, is acrolein (C_3_H_4_O), a strong irritant that affects the mucous membranes of the eye and respiratory tract and has a toxic effect on the heart and lungs (11, 16). When heated, propylene glycol can convert to a potent carcinogen, propylene oxide (C_3_H_6_O). Other carcinogens contained in heated vaping liquids, even those without nicotine, include propylene oxide and glycidol (17,18,19).

In addition to chemical breakdown products, high temperatures of heating elements (coils) can cause the transfer of heavy metals, such as nickel, cadmium, chromium, and lead, into the inhaled aerosol (20).

When evaluating the health risks of vape aerosol toxicants (such as formaldehyde, acrolein, propylene oxide, and glycidol), toxicological evaluation should take into account exposure and puff topography. Carbonyl formation during propylene glycol and vegetable glycerine pyrolysis is strongly non-linear and depends on heating temperatures. Under standard device operating conditions (low wattage and adequate e-liquid supply to the wick), thermal degradation remains relatively low. However, with high-power settings, including high coil temperatures (>200–250 °C) or “dry-puff” phenomena (heating an un-wicked coil), carbonyl emissions increase exponentially, occasionally reaching levels comparable to or exceeding occupational safety thresholds. However, while *in vitro* and animal models may demonstrate clear oxidative and cytotoxicity pathways, extrapolating these findings directly to human health requires caution due to differences in exposure duration, tissue architecture, and physiological metabolic clearance (21).

## IMPACT ON THE RESPIRATORY SYSTEM: FROM ACUTE INJURIES TO CHRONIC DISEASES

Inhaling vape aerosol poses an immediate threat to the respiratory system, manifesting as both acute and chronic conditions. Studies have shown that inhaled fine particles penetrate deep into the lungs, causing cell and DNA damage, inflammation, and scarring (22,23,24).

### E-cigarette or vaping-associated lung injury

In 2019, an outbreak of a severe acute lung illness was reported in the US, termed (with some minor variation) as e-cigarette or vaping-associated lung injury or EVALI. This condition is characterised by a sudden onset, and its symptoms include shortness of breath, cough, chest pain, fever, tachycardia, and gastrointestinal issues. According to the US Centers for Disease Control and Prevention (CDC), over 2,800 hospitalised cases were reported by February 2020, 68 of which were fatal (25). The primary driver of the EVALI epidemic was vitamin E acetate, a synthetic oil used as an illicit thickening agent in unregulated tetrahydrocannabinol (THC)-containing e-liquids (~84 % of documented cases). When inhaled, it penetrates the alveolar space, altering the surfactant phase behaviour and inducing severe parenchymal lipid pneumonia and diffuse alveolar damage (26,27,28).

### Other vaping-related lung diseases

In addition to EVALI, vaping is associated with a number of other serious respiratory diseases. The most common diseases and chemicals that trigger them are shown in Table 3. One of them is obliterative bronchiolitis, known as “popcorn lung”. This rare disease, affecting the small airways, is caused by the flavouring agent diacetyl, as it scars and narrows these passages until lung damage becomes irreversible (29, 30).

**Table 3:** Primary vaping-related pulmonary conditions and associated chemical aetiologies

**Disease / pathological condition**	**Pathogenetically related chemical(s)**	**Primary clinical manifestations & pathomorphology**	**Target airway structure**	**Ref.**
E-cigarette or vaping-associated lung injury (EVALI)	Vitamin E acetate (used with THC), thermal decomposition products	Acute shortness of breath, fever, hypoxia, diffuse alveolar damage, lipid-laden macrophages	Distal parenchyma and alveoli	(24, 26, 30)
Obliterative bronchiolitis (“Popcorn Lung”)	Diacetyl (flavouring agent)	Fibrotic scarring, luminal narrowing, irreversible airway obstruction	Small airways / bronchioles	(27)
Lipoid pneumonia	Exogenous oils / Fatty substances	Exogenous lipid accumulation, alveolar macrophage activation, localised parenchymal inflammation	Alveolar spaces	(28)
Primary spontaneous pneumothorax	Inhaled aerosol toxicants & pressure changes	Accumulation of air in the pleural cavity, subpleural bleb rupture, sudden lung collapse	Pleural space	(39)
Suppressed innate immune function	General vape aerosol components	Microfilament (actin) dysregulation, neutrophil immobilisation, impaired bacterial clearance	Pulmonary immune cells (neutrophils)	(31)

Other respiratory complications include lipoid pneumonia, which develops from inhaling fatty substances (oils) found in some vape liquids (31), and primary spontaneous pneumothorax, a dangerous condition characterised by the accumulation of air in the pleural cavity (32).

### Possible mechanisms compromising the lung immune system

Scientists at the University of Birmingham report that vaping can interfere with the normal function of immune cells, neutrophils in particular (33). The neutrophils they exposed to vape vapor remained viable but lost mobility, which rendered them unable to effectively protect the lungs from infections and chronic diseases. The cause of this phenomenon, report the authors, is the accumulation of filamentous actin and its polymerisation, preventing neutrophils to change shape and move to the site of injury. This mechanism is deeper than direct irritation and indicates profound systemic damage that may have long-term consequences.

## IMPACT ON THE CARDIOVASCULAR SYSTEM

### Acute effects of e-cigarettes on haemodynamic parameters

Nicotine in vape liquids rapidly enters the bloodstream through the lungs and stimulates the release of adrenaline, leading to an immediate increase in heart rate and blood pressure (34). These acute changes create excessive strain on the heart, especially in individuals who already have cardiovascular diseases. Furthermore, nicotine can cause cardiac arrhythmia, which has been observed even in young and healthy users (35).

While short- to medium-term transitions from combustible cigarettes to e-cigarettes may show modest haemodynamic improvements in younger cohorts, long-term cardiovascular risks remain a topic of active investigation. Observational cross-sectional population studies report a significant association between daily e-cigarette use and the prevalence of angina, myocardial infarction, and stroke (36, 37). However, these findings cannot confirm causality. Furthermore, these epidemiological datasets focus on exposure to nicotine-containing e-cigarettes, where nicotine-mediated sympathetic activation (tachycardia, elevated blood pressure) acts synergistically with aerosol-induced endothelial dysfunction and oxidative stress. In contrast, nicotine-free e-liquids do not induce these acute sympathomimetic haemodynamic spikes, though their thermal breakdown products (such as acrolein) continue to present potential vascular endothelial risks (38, 39).

### Chronic effects on the cardiovascular system

Unlike traditional smoking, which is often session-based, vapers can maintain a constant concentration of nicotine in their blood for an extended period. This continuous stimulation leads to a chronic state of vasoconstriction and elevated blood pressure contributing to cumulative damage and the development of serious diseases. Chronic e-cigarette use can also increase oxidative stress and systemic inflammation leading to the development of atherosclerosis (40), endothelial dysfunction, changes in blood serum composition, suppressed NO production, and increased microvascular permeability, which is a specific adverse outcome of vaping (41). E-cigarette users show a significant increase in the level of circulating proteins ICAM-1 and S100A8 in the blood serum, the first contributing to inflammation by recruiting leukocytes and the second to the activity of the receptor for advanced glycation end-products (RAGE), transendothelial migration, and vascular permeability (41).

Besides nicotine, some flavourings and other chemicals in the vape aerosol can cause inflammation in the walls of blood vessels, which increases the risk of clot formation that can lead to myocardial infarction or stroke (35, 36, 42).

## IMPACT ON THE NERVOUS SYSTEM AND COGNITIVE FUNCTION

### Specific vulnerability of the adolescent brain

Nicotine is a potent psychoactive substance that poses a particular threat to the developing brain, as its development continues until the age of 25. When adolescents vape, nicotine rapidly reaches their brain and binds to receptors responsible for releasing neurotransmitters such as dopamine, serotonin, and norepinephrine. This effect can disrupt the development of the prefrontal cortex, which is responsible for decision-making, impulse control, and executive functions (43), leading to impaired attention and learning. Nicotine also affects the hippocampus, impairing the ability to form new memories and retain information (44).

### Impact on mental health and addiction

Nicotine in vapes is one of the most highly addictive substances. Vaping can rapidly cause addiction, especially due to the high concentration of nicotine in salt liquids, which provides rapid saturation without a strong “throat hit.” The initial dopamine surge creates a fleeting sense of pleasure, but over time the brain adapts, requiring a larger dose to achieve a normal state. This can exacerbate feelings of depression and anxiety, making adolescents more dependent on nicotine to feel normal (45).

In addition, formaldehyde, contained in the aerosol, also affects the central nervous system, causing changes in brain functions that can lead to impaired memory, reduced learning ability, and sharp mood swings (46).

One study (47) conducted among university students showed that vapers demonstrated lower performance in learning, memory, problem-solving, and critical thinking tests compared to their non-vaping peers. The number of puffs per day directly correlated with a decrease in cognitive scores.

## VAPING COMPARED TO TRADITIONAL SMOKING: A CRITICAL ANALYSIS OF THE “HARM REDUCTION” CONCEPT

For a long time, the main argument in favour of vaping was the so called “harm reduction”, based on the assumption that vaping is significantly less harmful than traditional smoking (1,2,3). Proponents of this idea point to the absence of the combustion, which eliminates the formation of tars and other carcinogens associated with burning tobacco. They also note that vaping does not leave an unpleasant odour on clothes and hair, nor does it cause yellowing of teeth and nails, making it a more "aesthetic" alternative. Furthermore, vaping allows users to control their nicotine level, which theoretically could aid in the process of quitting addiction.

However, this “harm reduction” concept has increasingly been questioned. Available scientific evidence suggests that vaping is not less harmful but a different form of health risk with unique consequences (9, 10). A rigorous evaluation of vaping risks requires disentangling three distinct user cohorts: dual users, exclusive vapers, and complete switchers. Dual users smoke both combustible tobacco and e-cigarettes and experience the highest cumulative oxidative stress and biomarker levels, as vaping adds aerosol toxicants without eliminating combustion tars. The addition of vaping to conventional cigarette smoking multiplies the risk of developing lung cancer (48) as well as the overall health risk (49). Exclusive vapers, particularly adolescents who start vaping, experience novel biological risks including endothelial microvascular reactivity and bronchial inflammation (50). Smokers who completely replace combustible tobacco with e-cigarettes, in turn, exhibit substantial reductions in exposure to carcinogens such as 4-(methylnitrosamino)-1-(3-pyridyl)-1-butanol (NNAL) or benzene and improvements in acute vascular compliance (51). However, one systematic review (52) shows that smokers who switched to vaping after quitting traditional cigarettes run a higher risk of developing lung cancer than those who quit smoking entirely. In other words, vaping likely induces oncogenic processes through other, specific mechanisms, rather than those involving combustible tobacco, causing harm in a different way (20, 47) (Table 3).

## CONCLUSION AND DIRECTIONS FOR FUTURE RESEARCH

Vaping poses a serious and multifaceted threat to human health that extends far beyond nicotine addiction. The risks stem from the toxic composition of e-liquids that may contain nicotine and flavourings that decompose when heated into carcinogens and irritants, such as formaldehyde, acrolein, and propylene oxide, as well as heavy metals.

Systemic effects are many; vaping can adversely affect the respiratory system (causing diseases like EVALI and obliterative bronchiolitis), the cardiovascular system (by increasing blood pressure and causing chronic vasoconstriction), the nervous system (by disrupting adolescent brain development and causing cognitive impairment and mental disorders), and the immune system of the lungs (by rendering neutrophils immobile and less functional).

In terms of the advertised “harm reduction, new studies show that switching from cigarettes to vapes may increase the risk of developing lung cancer in former smokers.

Current knowledge about the long-term consequences of vaping is limited due to its relative novelty. Large-scale longitudinal studies are needed to better understand the cumulative effects of vaping over decades. Future research should also focus on the effects of various types of flavourings at the cellular and molecular level, the risks of “passive vaping”, especially in children, and the interaction between different chemical components and their synergistic effects.

## References

[1] Ramon-Torrell JM, Navarro-Zaragoza Javier (2025). New nicotine delivery devices and their role in smoking cessation and tobacco harm reduction strategies. Substance Abuse – New Compounds and New Problems.

[2] Micha JP, Rettenmaier MA, Bohart RD, Goldstein BH (2025). Vaping and smoking cessation. Subst Use Misuse.

[3] Ashour AM (2023). Use of vaping as a smoking cessation aid: a review of clinical trials. J Multidiscip Healthc.

[4] Becker TD, Rice TR (2022). Youth vaping: a review and update on global epidemiology, physical and behavioral health risks, and clinical considerations. Eur J Pediatr.

[5] Blank ML, Hoek J (2023). E-cigarette flavours and vaping as a social practice: implications for tobacco control. Crit Public Health.

[6] Abouzoor R, Al-Hamdani M (2025). Differences in vaping frequency and negative health effects experienced from vaping in a sample of vapers from three Middle Eastern countries. Heliyon.

[7] Irusa KF, Vence B, Donovan T (2020). Potential oral health effects of e-cigarettes and vaping: a review and case reports. J Esthet Restor Dent.

[8] Polosa R, Casale TB, Tashkin DP (2022). A close look at vaping in adolescents and young adults in the United States. J Allergy Clin Immunol Pract.

[9] Seiler-Ramadas R, Sandner I, Haider S, Grabovac I, Dorner TE (2021). Health effects of electronic cigarette (e-cigarette) use on organ systems and its implications for public health. Wien Klin Wochenschr.

[10] Hamann SL, Kungskulniti N, Charoenca N, Kasemsup V, Ruangkanchanasetr S, Jongkhajornpong P (2023). Electronic cigarette harms: aggregate evidence shows damage to biological systems. Int J Environ Res Public Health.

[11] Sun Y, Lin K, Effah F, Barrera FC, Li D, McIntosh S, McGraw MD, Rahman I (2025). Toxicity of humectants propylene glycol and vegetable glycerin in electronic nicotine delivery systems. Toxicol Lett.

[12] Prochaska JJ, Vogel EA, Benowitz N (2022). Nicotine delivery and cigarette equivalents from vaping a JUULpod. Tob Control.

[13] Mandalika MA, Fawzy A (2024). Be careful, ladies! The potential damaging impact of vaping and electric cigarettes for healing of post-caesarean-section wounds: a literature review. Int J Med Sci Clin Res Studies.

[14] Vyshneva V (2022). Effect of propylene glycol and vegetable glycerine ratio in e-liquid on aerosol formation: overview of relevant properties. Addictology.

[15] Tong Y, Xiong Y, Yan Q, Gao S, Le X, Wei P, Shu H, Wang Z, Tang X, Li P, Xiong Z, Wang Y (2021). Effects of glycerol and propylene glycol on smoke release of heat-not-burn tobacco products. J Phys: Conf Ser.

[16] Khatun J, Dhak D (2025). Emerging acrolein: a pulmonary and cardiac hazard and its association with cigarette, and e-cigarettes. Chem Biol Interact.

[17] Cui L, Zheng Y, Ma W, Han S, Wang M, Liu Z, Wang J, Song S, Chen H, Hou H, Hu Q (2025). Assessment and characterization of emerging contaminants in aerosols from novel tobacco products: emphasis on glycidol, chloropropanol, and potential exposure biomarkers. Microchem J.

[18] Lorkiewicz P, Keith R, Lynch J, Jin L, Theis W, Krivokhizhina T, Riggs D, Bhatnagar A, Srivastava S, Conklin DJ (2022). Electronic cigarette solvents, JUUL E-liquids, and biomarkers of exposure: in vivo evidence for acrolein and glycidol in e-cig-derived aerosols. Chem Res Toxicol.

[19] Li Y, Burns AE, Tran LN, Abellar KA, Poindexter M, Li X, Madl AK, Pinkerton KE, Nguyen TB (2021). Impact of e-liquid composition, coil temperature, and puff topography on the aerosol chemistry of electronic cigarettes. Chem Res Toxicol.

[20] Shehata SA, Toraih EA, Ismail EA, Hagras AM, Elmorsy E, Fawzy MS (2023). Vaping, environmental toxicants exposure, and lung cancer risk. Cancers (Basel).

[21] Soulet S, Sussman RA (2022). Critical review of the recent literature on organic byproducts in e-cigarette aerosol emissions. Toxics.

[22] Auschwitz E, Almeda J, Andl CD (2023). Mechanisms of e-cigarette vape-induced epithelial cell damage. Cells.

[23] Park J-A, Crotty Alexander LE, Christiani DC (2022). Vaping and lung inflammation and injury. Annu Rev Physiol.

[24] Masso-Silva JA, Byun MK, Crotty Alexander LE (2021). Acute and chronic effects of vaping electronic devices on lung physiology and inflammation. Curr Opin Physiol.

[25] Centers for Disease Control and Prevention (CDC) (2020). Outbreak of lung injury associated with e-cigarette use, or vaping products [displayed 9 September 2026].

[26] Kalininskiy A, Bach CT, Nacca NE, Ginsberg G, Marraffa J, Navarette KA, McGraw MD, Croft DP (2019). E-cigarette, or vaping, product use associated lung injury (EVALI): case series and diagnostic approach. Lancet Respir Med.

[27] Kazachkov M, Pirzada M (2020). Diagnosis of EVALI in the COVID-19 era. Lancet Respir Med.

[28] Soto B, Costanzo L, Puskoor A, Akkari N, Geraghty P (2023). The implications of vitamin E acetate in e-cigarette, or vaping, product use-associated lung injury. Ann Thorac Med.

[29] Gaur R, Ram G (2021). Popcorn lung: the e-disease. Int J Pharm Chem Biol Sci.

[30] Esquer C, Echeagaray O, Firouzi F, Savko C, Shain G, Bose P, Rieder A, Rokaw S, Witon-Paulo A, Gude N, Sussman MA (2022). Fundamentals of vaping-associated pulmonary injury leading to severe respiratory distress. Life Sci Alliance.

[31] Alsuwayah M, Abuserewa ST, Test V, Nugent KM (2025). A Case of vaping induced lipoid pneumonia. Am J Respir Crit Care Med.

[32] Al-Taj M, Alsabbah A, Ma'ali T, Abu Suilik M, AlSamhori JF, Alloubani A, Madha A, Goyal AV, Gharaibeh A (2025). Vaping-associated pneumothorax: a systematic review of case reports and case series. Medicina (Kaunas).

[33] Jasper AE, Faniyi AA, Davis LC, Grudzinska FS, Halston R, Hazeldine J, Parekh D, Sapey E, Thickett DR, Scott A (2024). E-cigarette vapor renders neutrophils dysfunctional due to filamentous actin accumulation. J Allergy Clin Immunol.

[34] Poudel R, Li S, Hong H, Zhao J, Srivastava S, Robertson RM, Hall JL, Srivastava S, Hamburg NM, Bhatnagar A, Keith RJ (2024). Catecholamine levels with use of electronic and combustible cigarettes. Tob Induc Dis.

[35] Rose JJ, Krishnan-Sarin S, Exil VJ, Hamburg NM, Fetterman JL, Ichinose F, Perez-Pinzon MA, Rezk-Hanna M, Williamson E, American Heart Association Council on Cardiopulmonary, Critical Care, Perioperative and Resuscitation, Council on Epidemiology and Prevention, Council on Cardiovascular Radiology and Intervention, Council on Lifestyle and Cardiometabolic Health, Council on Peripheral Vascular Disease, Stroke Council, Council on Arteriosclerosis, Thrombosis and Vascular Biology (2023). Cardiopulmonary impact of electronic cigarettes and vaping products: a scientific statement from the American Heart Association. Circulation.

[36] Meng X-c, Guo X-x, Peng Z-y, Wang C, Liu R (2023). Acute effects of electronic cigarettes on vascular endothelial function: a systematic review and meta-analysis of randomized controlled trials. Eur J Prev Cardiol.

[37] Nagy S, Abdool A, Rocco O, Shirazi N, Israilov D, Atiquzzaman N, Amini K, Kesselman MM (2025). The impacts of vaping on the cardiovascular system: a systematic review of case reports. Cureus.

[38] Kundu A, Feore A, Sanchez S, Abu-Zarour N, Sutton M, Sachdeva K, Seth S, Schwartz R, Chaiton M (2025). Cardiovascular health effects of vaping e-cigarettes: a systematic review and meta-analysis. Heart.

[39] Rezk-Hanna M, Rossman MJ, Ludwig K, Sakti P, Cheng C-W, Brecht M-L, Benowitz NL, Seals DR (2024). Electronic hookah (waterpipe) vaping reduces vascular endothelial function: the role of nicotine. Am J Physiol Heart Circ Physiol.

[40] Magna A, Polisena N, Polisena L, Bagnato C, Pacella E, Carnevale R, Nocella C, Loffredo L (2024). The hidden dangers: e-cigarettes, heated tobacco, and their impact on oxidative stress and atherosclerosis – a systematic review and narrative synthesis of the evidence. Antioxidants (Basel).

[41] Mohammadi L, Han DD, Xu F, Huang A, Derakhshandeh R, Rao P, Whitlatch A, Cheng J, Keith RJ, Hamburg NM, Ganz P, Hellman J, Schick SF, Springer ML (2022). Chronic e-cigarette use impairs endothelial function on the physiological and cellular levels. Arterioscler Thromb Vasc Biol.

[42] Lyytinen G, Brynedal A, Anesäter E, Antoniewicz L, Blomberg A, Wallén H, Bosson JA, Hedman L, Mobarrez F, Tehrani S, Lundbäck M (2023). Electronic cigarette vaping with nicotine causes increased thrombogenicity and impaired microvascular function in healthy volunteers: a randomised clinical trial. Cardiovasc Toxicol.

[43] Adams D, Kaliss N, Missner A, Valentine MM (2021). The interplay of nicotine and social stress mediate dopaminergic neuron firing in the ventral tegmental area-nucleus accumbens pathway, contributing to stress and depressive mood disorders. Georgetown Sci Res J.

[44] Youssef M (2025). Nicotine e-cigarette exposure in adolescence induces long-term cognitive and region-specific alterations in the hippocampus and prefrontal cortex.

[45] Murray L, Scavnicky MK, Korponay C, Lukas SE, Frederick BB, Janes AC (2025). Brain reactivity to nicotine cues mediates the link between resting-state connectivity and cue-induced craving in individuals who smoke or vape nicotine. Neuropsychopharmacology.

[46] López-Ojeda W, Hurley RA (2024). Vaping and the brain: effects of electronic cigarettes and e-liquid substances. J Neuropsychiatry Clin Neurosci.

[47] Hashim NM, Kamarudin SN, Daud AZC, Ahmedy F, Salim MS, Hassan SAA (2023). Impact of e-cigarette usage on cognitive performance level among university students in malaysia: a case-control study. Southeast Asian J Trop Med Public Health.

[48] Pisinger C, Rasmussen SKB (2022). The health effects of real-world dual use of electronic and conventional cigarettes versus the health effects of exclusive smoking of conventional cigarettes: a systematic review. Int J Environ Res Public Health.

[49] Sompa S.I., Ji J., Rahman M, Sjögren B, Upadhyay S, Ganguly K, Olin AC, Bergström A, Palmberg L (2025). Local and systemic effects in e-cigarette users compared to cigarette smokers, dual users, and non-smokers. Respir Res.

[50] Boakye E, Uddin SMI, Osuji N, Meinert J, Obisesan OH, Mirbolouk M, Tasdighi E, El-Shahawy O, Erhabor J, Osei AD, Rajan T, Patatanian M, Holbrook JT, Bhatnagar A, Biswal SS, Blaha MJ (2023). Examining the association of habitual e-cigarette use with inflammation and endothelial dysfunction in young adults: The VAPORS-Endothelial function study. Tob Induc Dis.

[51] Dai H, Benowitz NL, Achutan C, Farazi PA, Degarege A, Khan AS (2022). Exposure to toxicants associated with use and transitions between cigarettes, e-cigarettes, and no tobacco. JAMA Netw Open.

[52] Mohapatra S, Tiwari A, Kandala A, Winayak R, Ghose A, Das R, Hasanova M, Gunani M, Jaswal G, Mitra S, Noronha V, Jain A, Merletti F, Passaro A, Cortellini A, Addeo A, Banna GL, Boussios S (2025). The risk of lung cancer from vaping or e-cigarette usage: a systematic review. ESMO Open.

